# Regulation of miRNA Transcription in Macrophages in Response to *Candida albicans*

**DOI:** 10.1371/journal.pone.0013669

**Published:** 2010-10-27

**Authors:** Claire E. Monk, György Hutvagner, J. Simon C. Arthur

**Affiliations:** 1 MRC Protein Phosphorylation Unit, College of Life Sciences, Sir James Black Complex, University of Dundee, Dundee, Scotland; 2 Wellcome Trust Centre for Gene Regulation and Expression, College of Life Sciences, Sir James Black Complex, University of Dundee, Dundee, Scotland; Ludwig-Maximilians-Universität München, Germany

## Abstract

Macrophages detect pathogens via pattern recognition receptors (PRRs), which trigger several intracellular signaling cascades including the MAPK and NFκB pathways. These in turn mediate the up-regulation of pro-inflammatory cytokines that are essential to combat the pathogen. However as the over-production of pro-inflammatory cytokines results in tissue damage or septic shock, precise control of these signaling pathways is essential and achieved via the induction of multiple negative feedback mechanisms. miRNAs are small regulatory RNAs that are able to affect protein expression, via the regulation of either mRNA stability or translation. Up-regulation of specific miRNAs could have the potential to modulate PRR signaling, as has been shown for both miR-146 and miR-155. Here we have analysed which miRNAs are up-regulated in mouse macrophages in response to the fungal pathogen heat killed *Candida albicans* and compared the profile to that obtained with the TLR4 ligand LPS. We found that in addition to miR-146 and miR-155, both *Candida albicans* and LPS were also able to up-regulate miR-455 and miR-125a. Analysis of the signaling pathways required showed that NFκB was necessary for the transcription of all 4 pri-miRNAs, while the ERK1/2 and p38 MAPK pathways were also required for pri-miR-125a transcription. In addition the anti-inflammatory cytokine IL-10 was found to be able to induce miR-146a and b, but inhibited miR-155 induction. These results suggest that miR-455, miR-125, miR-146 and miR-155 may play important roles in regulating macrophage function following PRR stimulation.

## Introduction

The innate immune system is able to detect infection via the recognition of various pathogen specific molecules, referred to as pathogen derived molecular patterns or PAMPs. Recognition of PAMPs occurs via specific groups of ‘pathogen recognition receptors’ or PRRs, including Toll-like receptors (TLRs), Nod-like receptors, CARD domain helicases such as RIG-I and C-type lectins such as dectin-1. Each of these receptors is specific for certain PAMPs, and therefore different pathogens will be sensed by different combinations of PRRs (reviewed in [1], [2], [3]). These differences allow for the fine-tuning of the immune response to the type of invading pathogen. An important consequence of the activation of cells in the innate immune system is the production of pro-inflammatory cytokines, which help co-ordinate the immune response and promote inflammation at the site of infection. While pro-inflammatory cytokines are critical to combat pathogens, excess or inappropriate production of pro-inflammatory cytokines has serious consequences including tissue damage and septic shock. In addition, excess production of inflammatory cytokines contributes to a number of pathologies including auto-inflammatory and autoimmune disorders. Precise control of innate immune cells and their production of pro-inflammatory cytokines is therefore critical. In addition to the positive signals provided by the pathogens and pro-inflammatory cytokines themselves, multiple inhibitory feedback mechanisms also act on inflammatory cytokine production, including direct negative feedback mechanisms in PRR activated signaling cascades and the production of anti-inflammatory cytokines such as IL-10.

Recently, it has been suggested that an additional control mechanism in activated macrophages is the induction of specific miRNAs. miRNAs are small regulatory RNAs of 21 to 24 bp in length that have been shown to modulate several processes including development, immunity and neuronal function. In mammalian cells, miRNAs predominantly regulate protein expression at a post-transcriptional level by repressing the translation of their target mRNAs (reviewed in [4], [5]). In the genome, miRNAs can be located in the introns of protein coding and non-coding genes, the exons of non-coding genes and in intragenic regions. miRNAs are initially transcribed and processed to give rise to a primary miRNA (pri-miRNA). The majority of pri-miRNAs are then further processed in the nucleus by the Microprocessor complex to release a hairpin structured pre-miRNA. The pre-miRNAs are next exported into the cytoplasm where they are further processed by Dicer into a small double stranded intermediate. One strand of a miRNA is then loaded into RISC (RNA Induced Silencing Complex) of which a key component is an Argonaute protein.

Previous studies have shown that the transcription of some miRNA genes can be modulated by the activation of TLRs in the innate immune system. In THP-1 cells, the transcription of miR-146a/b, miR-132, and miR-155 was found to be up-regulated by LPS stimulation [6]. Interestingly, in this study it was found that miR-146 was more strongly induced by TLRs located in the plasma membranes compared to endosomal TLRs. As endosomal TLRs are important to sense viral nucleic acid, this could suggest that miRNAs may help tailor the immune response to the type of invading pathogen [6]. More recent reports have however shown that miR-146 can be induced by viral stimuli [7], [8], [9]. Murine macrophages have also been shown to induce miR-155 in response to the TLR4 agonist LPS or the TLR3 agonist polyI:C [10], [11]. LPS has been shown to down-regulate the expression of miR-125b in the Raw macrophage cell line [10], however it was found to be up-regulated in a cholangiocyte cell line in response to LPS or *Cryptossporidium parvum* infection [12].

The most likely function of TLR induced miRNAs is to act as a negative feedback mechanism to inhibit TLR mediated signaling. For instance, miR-146 can target IRAK1 and Traf6, key components in linking TLRs to their downstream signaling cascades [6], [8], as well as STAT1 and IRF5, transcription factors implicated in pro-inflammatory cytokine production [9]. miR-155 has been shown to target SHIP1, which can affect macrophage function by modulating PI-3K dependent signaling, C/EBPβ (an important transcription factor in the inflammatory response), and the TLR adaptor protein Myd88 [13], [14], [15].

While considerable effort has been made to understand how bacteria and viruses activate the innate immune system, the response to fungal ligands is less well understood. While in healthy individuals fungal infections are usually mild, in immuno-compromised patients these infections are much more serious and can be a significant cause of mortality in this group. Because of the increasing number of immuno-compromised patients due to diseases such as HIV or immunosuppressive therapies for conditions such as cancer or autoimmunity this is an increasing clinical problem. Given the potential importance of miRNAs in the innate immune response, we carried out a comparison of the ability of a fungal PAMP, heat killed *Candida albicans*, and LPS, a component of Gram –ve bacterial cell walls, to induce miRNA genes and examined the signaling pathways that regulate their transcription.

## Results

To determine if fungal ligands were able to up-regulate miRNAs, murine bone marrow derived macrophages (BMDMs) were stimulated with heat killed *C. albicans* for 16 hours. RNA was isolated from both un-stimulated and stimulated cells and miRNA expression profiled on arrays (LC Biosciences). The experiment was performed in triplicate using independent cultures from three different mice. Of the 617 miRNAs on the chip, 366 were detected on at least 2 of the 6 arrays performed. Analysis of the relative expression of these miRNAs before and after stimulation with heat killed *C. albicans* showed that the expression of most of these was unaffected by treatment. Only 2 miRNAs, miR-155 and miR-455, were more than 2-fold up-regulated with a p-value of less than 0.05 (Table 1). A parallel experiment using LPS as a stimulus also gave relatively small numbers of significantly up-regulated miRNAs. With LPS only 4 miRNAs, miR-155, miR-125-5p and 3p and miR-146a, showed greater than 2-fold up-regulation on the two arrays with a *p* value of less than 0.05 (Table 2). Both miR-146b, which has previously been shown to be up-regulated in macrophages and miR-455 were also up-regulated in this experiment, however their *p* values were 0.16 and 0.10 respectively.

**Table 1:** miRNAs up-regulated by heat killed *Candida albicans* treatment of BMDMs.

miRNA ID	Fold induction	*p* value
mmu-miR-155	157.59	0.005
mmu-miR-455	3.01	0.009
mmu-miR-148b	1.71	0.019
mmu-miR-135a*	1.68	0.049
mmu-miR-125a-5p	1.60	0.063
mmu-miR-146a	1.56	0.097
mmu-miR-125a-3p	1.54	0.092
mmu-miR-192	1.52	0.061
mmu-miR-192	1.52	0.061
mmu-miR-139-5p	1.5	0.042

Microarray analysis was carried out by LC Sciences on BMDMs either treated with heat killed *C. albicans* for 16 h or left untreated. Average fold induction and *p* values were obtained from analysis of triplicate experiments. The miRNAs that showed a fold change greater than 1.5 and *p* value of <0.01 are represented.

**Table 2:** miRNAs up-regulated by LPS treatment of BMDMs.

miRNA ID	Fold induction	*p* value
mmu-miR-155	229.13	0.047
mmu-miR-125a-3p	7.89	0.095
mmu-miR-125a-5p	2.58	0.066
mmu-miR-146a	2.25	0.042
mmu-miR-99b	1.61	0.043

Microarray analysis was carried out by LC Sciences on BMDMs either treated with heat killed *C. albicans* for 16 h or left untreated. Average fold induction and *p* values were obtained from analysis of duplicate experiments. The miRNAs that showed a fold change greater than 1.5 and *p* value of <0.01 are represented.

**Figure 1:**
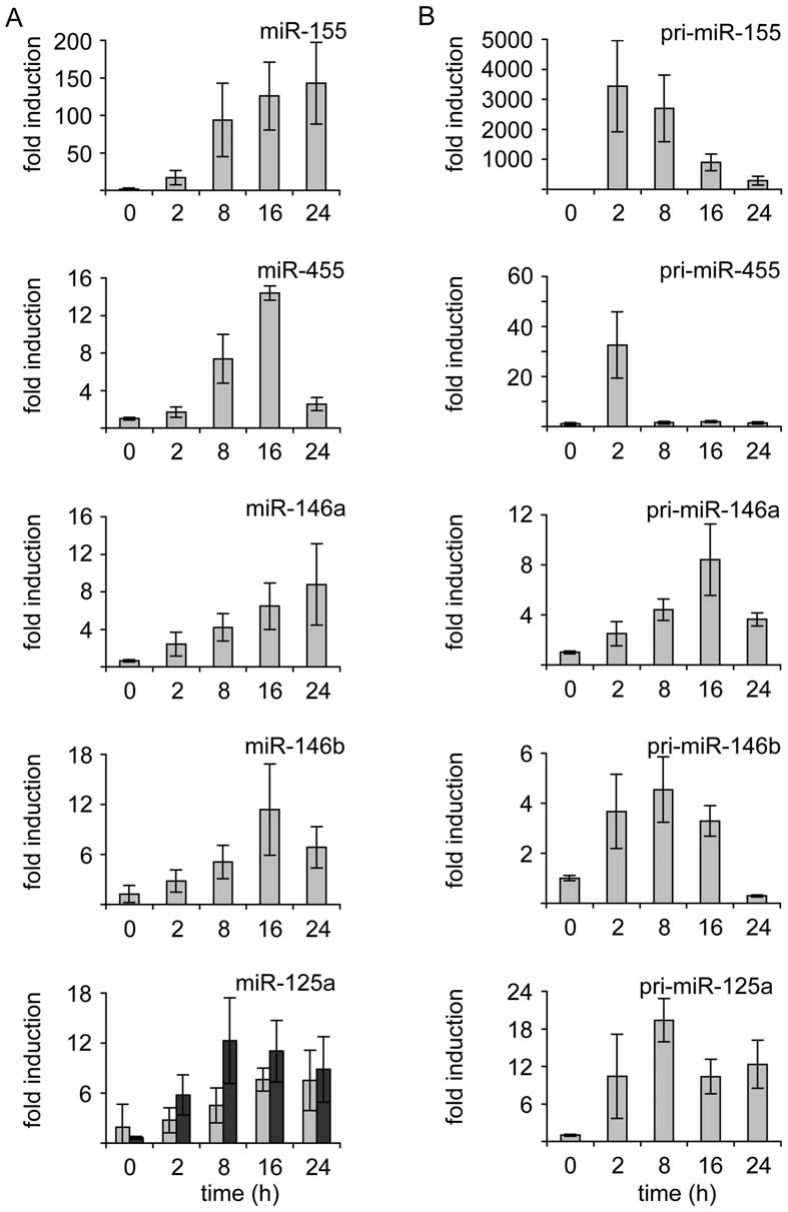
LPS stimulates the transcription of miR-155, miR-455, miR-146 and miR-125a. BMDMs were isolated from C57/Bl6 mice. Cells were stimulated with 100 ng/ml of LPS for the indicated times. Cells were then lysed and total RNA isolated as described in the methods. (A) The levels of miR-155, miR-455, miR-146a, miR-146b, miR-125a-5p (grey bars) and miR-125a-3p (black bars) were determined by a Taqman based Q-PCR (Applied Biosystems). 18S levels was used to normalise the amount of RNA in the reaction. (B) The levels of pri-miR-155, pri-miR-455, pri-miR-146a, pri-miR-146b and pri-miR-125a were determined by Q-PCR. 18S was used as a loading control. Error bars represent the standard deviation of independent stimulations cultures from 4 mice.

To extend the results from the array experiment, the transcriptional regulation of miR-125-5p and 3p, miR-155, miR-146a, miR-146b and miR-455 by either heat killed *C. albicans* or LPS was examined using Taqman qPCR based methods, in samples from BMDMs generated independently from those used for the array studies. Stimulation of BMDMs with LPS resulted in a gradual increase over 24 h in the levels of mature miR-125a-3p and 5p, miR,-146a, mir-146b and miR-155 following LPS stimulation (Fig. 1A). Consistent with this, up-regulation of the primary transcripts was also seen for these miRNAs in response to LPS, although this was more rapid for the pri-miRNA transcripts than for the mature miRNA (Fig. 1B). Both pri- and mature miR-455 were also up-regulated by LPS, however in contrast to the other miRNAs tested the up-regulation of the mature miR-455 was faster and more transient than for the other pri-miRNAs tested (Fig. 1A & B).

**Figure 2:**
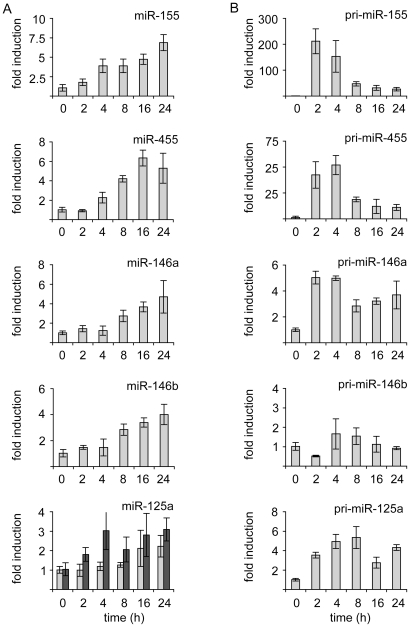
Heat killed *Candida albicans* stimulates the transcription of miR-155, miR-455, miR-146 and miR-125a. BMDMs were isolated from C57/Bl6 mice. Cells were stimulated with 10^6^ cells/ml of heat killed *C. albicans* for the indicated times. Cells were then lysed and total RNA isolated as described in the methods. (A) The levels of miR-155, miR-455, miR-146a, miR-146b, miR-125a-5p (grey bars) and miR-125a-3p (black bars) were determined by a Taqman based Q-PCR (Applied Biosystems). 18S levels were used to normalise the amount of RNA in the reaction. (B) The levels of pri-miR-155, pri-miR-455, pri-miR-146a, pri-miR-146b and pri-miR-125a were determined by Q-PCR. 18S was used as a loading control. Error bars represent the standard deviation of independent stimulations cultures from 4 mice.

In response to heat killed *C. albicans* miR-155 and miR-455 were the most highly up-regulated of the miRNAs tested. However, while the fold up-regulation of miR-455 was similar between LPS and heat killed *C. albicans*, LPS gave a much higher fold stimulation of miR-155 than heat killed *C. albicans* (150 compared to 7 fold). Heat killed *C. albicans* also caused a modest up-regulation of miR-125a, mirR-146a and miR-146b (Fig. 2A). The levels of pri-miR-146a and pri-miR-125a were also increased by heat killed *C. albicans* treatment. While there was a trend for a small increase in pri-miR-146b, this did not reach statistical significance (p>0.05, 2 tailed students *t*-test).

**Figure 3:**
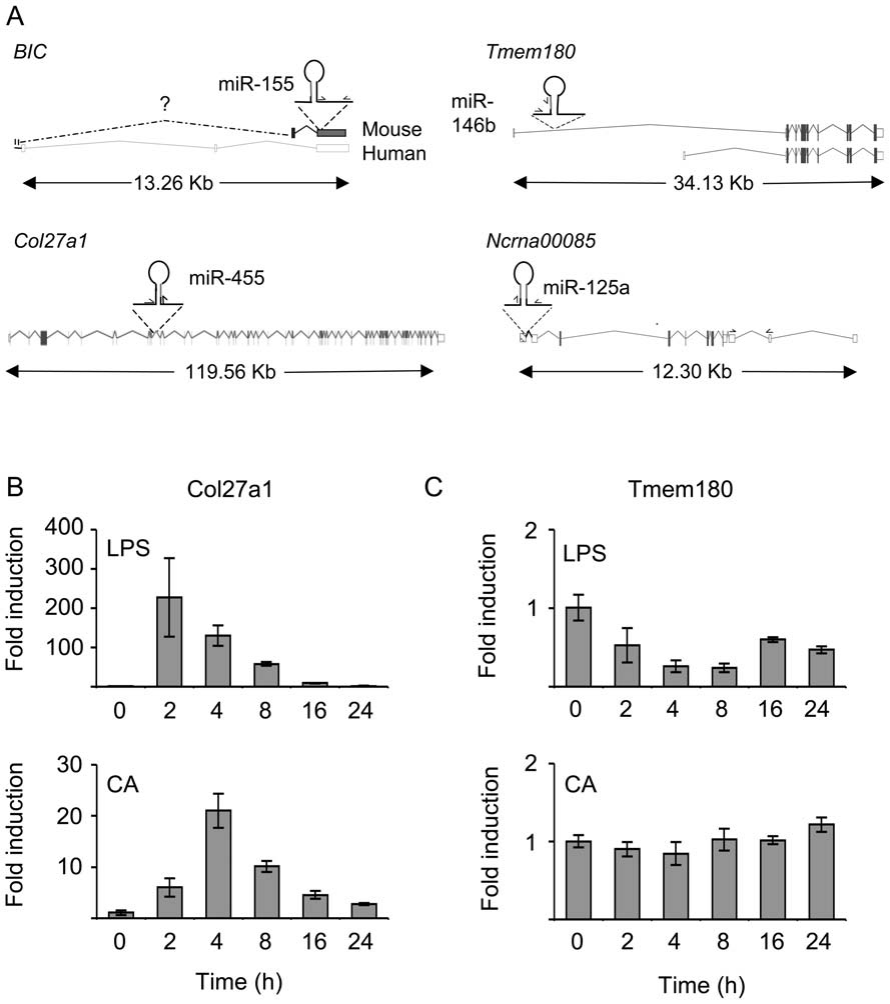
miR-146b, mir-155, miR-455 and miR-125a are located in annotated genes. (A) The locations of the miRNA are shown relative to the genes in which they are contained. The structure of the genomic loci are shown, with coding exons shown by filled boxes and non-coding exons by open boxes. miR-155 is located in the exon of the non-protein coding BIC gene, while miR-146b and miR-455 are encoded in the introns of Tmem180 and Col27a1 respectively. miR-125a is located 5′ to the Ncrna00085 transcript. ESTs suggest that a further exon (hatched box), containing miR-125a, exists upstream of Ncrna00085. (B) BMDMs were stimulated with 100 ng/ml of LPS (upper panel) or 10^6^ cells/ml heat killed *C. albicans* (CA, lower panel) for the indicated times. Cells were then lysed and total RNA isolated as described in the methods. The levels of mRNA for Col27a1 were determined by Q-PCR. Error bars represent the standard deviation of stimulations from 4 independent cultures. (C) As (B) except that the levels of Tmem180 using primers spanning exons 3 to 4 were measured.

**Figure 4:**
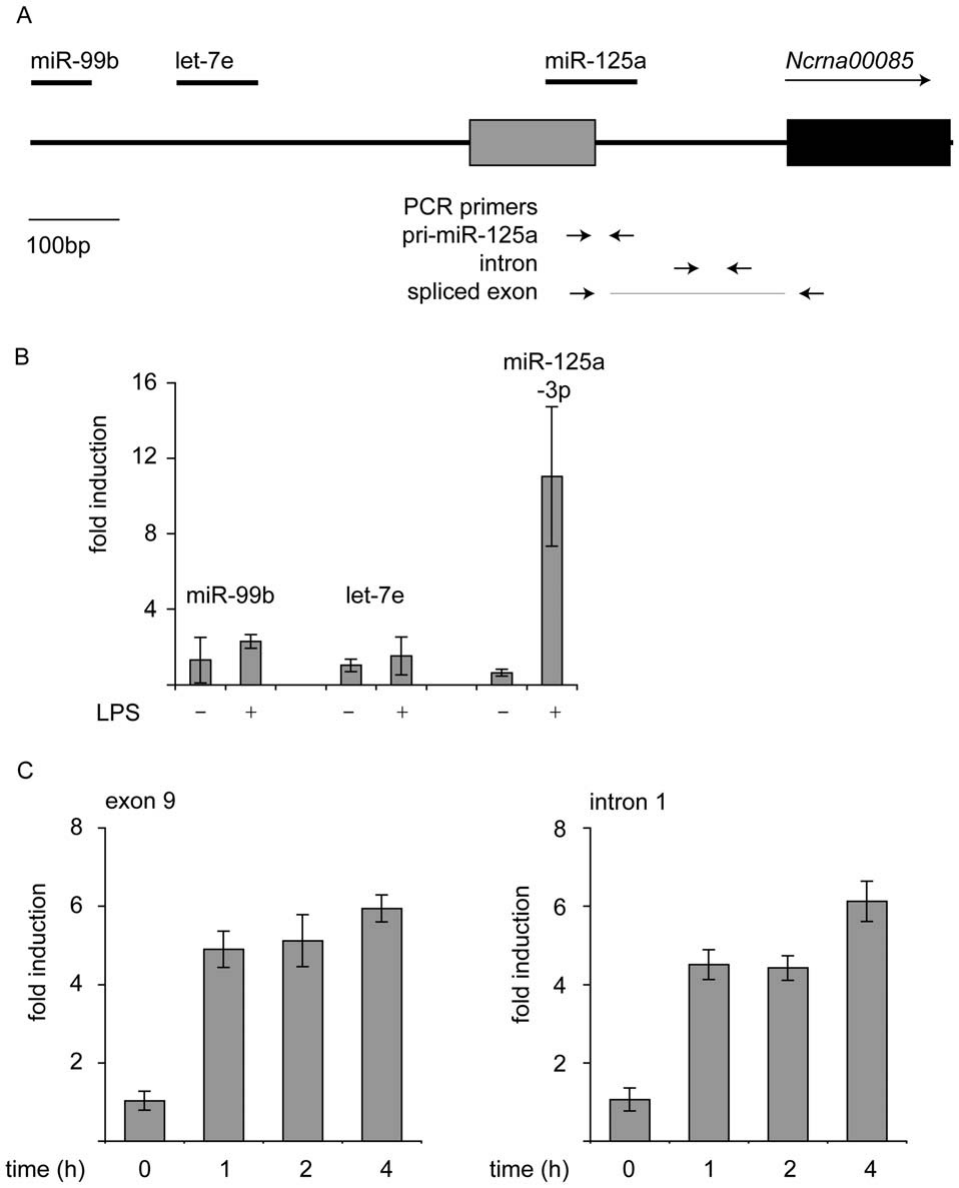
Regulation of the miR-125a locus. (A) A schematic diagram of the genomic locus encoding miR-125a is shown. Pri-mir-125a is located upstream of the 1^st^ exon of Ncrna00085 (black box), overlapping with an EST (CA465266, grey box). Two further miRNAs, miR-99b and let-7e. The position of the primer sets used to amplify regions of this locus are indicated. (B) BMDMs were stimulated with 100 ng/ml LPS for 16 h. Total RNA was isolated and the levels of mirR-99b, let-7e and miR-125a-3p determined by Q-PCR. Error bars represent the standard deviation from 4 independent cultures. (C) BMDMs were stimulated with 100 ng/ml LPS for the indicated times. Total RNA was isolated and the levels Ncrna00085 intron 1 and exon 9 determined by Q-PCR. Error bars represent the standard deviation from 4 independent cultures.

Of the miRNAs examined, miR-146a is located in an intragenic region, while the others are encoded within or adjacent to annotated genes (Fig. 3A). miR-455 is encoded in intron 10 of the Col27a1 gene. Similar to miR-455, Col27a1 mRNA transcription was induced by both LPS and heat killed *C. albicans* (Fig. 3B). miR-146b is potentially located within the Tmem180 gene in mice. Two alternate transcriptional start sites, resulting in two alternate 1^st^ exons, have been annotated for this gene. miR-146b is located in the 1^st^ intron downstream of the distal 1^st^ exon (Fig. 3A). Analysis of Tmem180 mRNA levels with primers located in exon 4 indicated that this gene was transiently repressed in response to LPS but not affected by heat killed *C. albicans*, suggesting that miR-146b is transcribed independently to Tmem180 in macrophages (Fig. 3C). miR-125a is located in the 5′ region of a putative protein-encoding gene, Ncrna00085 (Fig. 3A). While several potential 5′ exons are predicted for Ncrna00085, miR-125a was found to span the 3′ splice site of the 1^st^ annotated exon. In addition two further miRNAs, miR-99b and let-7e are located within a 1kb region upstream of miR-125a (Fig. 4A). In contrast to miR-125a, LPS did not up-regulate the levels of either miR-99b or let-7e (Fig. 4B), suggesting that they were regulated independently of miR-125a. RT-PCR with primers in the potential 1^st^ exon and 2^nd^ exon of Ncrna00085 was able to amplify a product, which on sequencing was found to correspond to the expected mRNA sequence for splicing of these exons (data not shown). Q-PCR with these primers, or with primers internal to either the 1^st^ intron or exon 9 of Ncrna00085 showed that Ncrna00085 was induced in response to either LPS or heat killed *C. albicans* (Fig. 4C, data not shown).

### MAPK signaling contributes to miRNA up-regulation in macrophages

**Figure 5:**
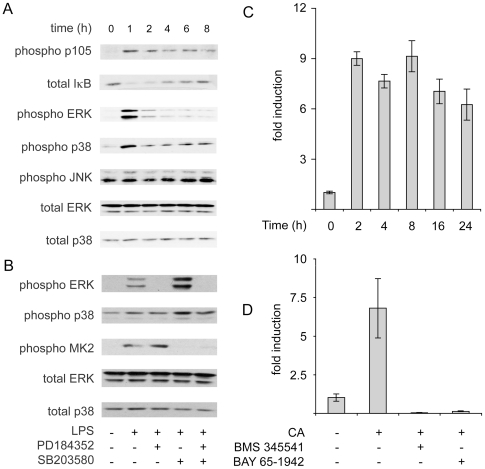
Heat killed *Candida albicans* stimulates ERK1/2, p38 and NFκB. (A) BMDMs were stimulated for the indicated times with 10^6^ cells/ml heat killed *C. albicans*. Cells were lsyed in SDS sample buffer and the levels of phospho ERK1/2, total ERK1/2, phospho p38, total p38, phospho JNK, phospho p105 and total IκB were determined by immunoblotting. (B) BMDMs were incubated with 2 µM PD184352 or 5 µM SB203580 as indicated for 60 min prior to stimulation with 10^6^ cells/ml heat killed *C. albicans* for 60 min. Cells were lsyed in SDS sample buffer and the levels of phospho ERK1/2, total ERK1/2, phospho p38, total p38 and phospho MK2 were determined by immunoblotting. (C) BMDMs were stimulated with 10^6^ cells/ml heat killed *C. albicans*. Total RNA was isolated and the levels of IκBα mRNA determined by Q-PCR. Error bars represent the standard deviation from 4 independent cultures. (D) Where indicated BMDMs were treated with 15 µM BMS 345541 or 10 µM BAY 65-1942 for 1 h. Cells were then stimulated with 10^6^ cells/ml heat killed *C. albicans* for 4 h, and IκB levels determined by Q-PCR. Error bars represent the standard deviation from 4 independent cultures.

In macrophages, both the MAPK and NFκB signaling pathways are known to be important for the transcriptional regulation of many genes that are induced in response to PAMPs. LPS is well established to activate the p38, ERK and JNK MAPK signaling cascades as well as the canonical NFκB pathway. In BMDMs, heat killed *C. albicans* was also able to activate the ERK1/2 and p38 MAPK pathways, as shown by immunoblotting for phosphorylation of the TXY motif in the MAP kinase activation loop (Fig. 5A–B). Little or no activation of JNK was observed following treatment with heat killed *C. albicans*. NFκB signaling was also activated by heat killed *C. albicans* as indicated by the loss of the inhibitory IκB protein and phosphorylation of the p105 NFκB subunit (Fig. 5A). Additionally, Q-PCR also showed that transcription of the NFκB dependent gene IκBα was stimulated by heat killed *C. albicans* (Fig. 5C). The induction of IκBα mRNA was blocked by the IKKβ inhibitors BMS-345541 [16] or BAY65-1942 [17] (Fig. 5D).

**Figure 6:**
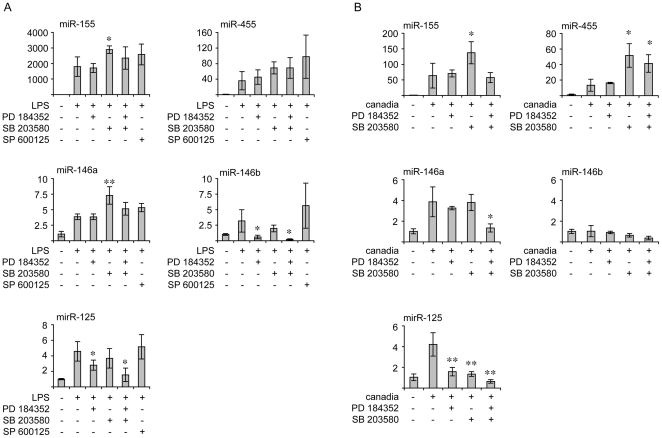
Effect of MAPK inhibitors on the transcription of miR-155, miR-455, miR-146 and miR-125a. (A) BMDMs were treated for 1 h where indicated with 2 µM PD 184352, 5 µM SB203580 or 10 µM SP 600125. Cells were then stimulated with 100 ng/ml LPS for 1 h and total RNA isolated. The levels of pri-miR-155, pri-miR-455, pri-miR-146a, pri-miR-146b and pri-miR-125a were determined by Q-PCR. 18S levels were used as a loading control. Error bars represent the standard deviation of independent stimulations on cultures from 4 mice. (B) As (A) except that cells were pretreated for 1 h with 2 µM PD 184352 or 5 µM SB203580 were indicated and then stimulated with 10^6^ cells/ml heat killed *C. albicans* for 4 h. For comparison of LPS or *C. albicans* alone relative to inhibitor treated conditions, *p* values of less than 0.05 are indicated by * and less than 0.01 by ** (students *t*-test).

To examine the involvement of MAPK signaling in the transcription of the selected miRNAs, BMDMs were pretreated with PD184352, an inhibitor of MEK1/2 that blocks ERK1/2 activation or SB203580, a p38α/β inhibitor [18]. PD184352 has been previously shown to block LPS induced ERK1/2 activation [34] and is also able to block ERK1/2 phosphorylation in response to heat killed *C. albicans* (Fig. 5B). Similarly SB203580 was able to inhibit the phosphorylation of the p38α substrate MK2 following heat killed *C. albicans* stimulation (Fig. 5B). BMDMs were also pre-treated with SP600125, a compound that inhibits JNK. SP600125 is however not completely specific for JNK and can inhibit several other protein kinases [18]. It is therefore difficult to use SP600125 to demonstrate a role for JNK, however the lack of an effect of SP600125 can be used to exclude a role for JNK. As SP600125 did not significantly affect any of the miRNAs tested, this would suggest that their transcription is independent of the JNK pathway. The induction of pri-miR-155, pri-miR-455 and pri-miR-146a was not reduced by pre-treatment with PD184352, SB203580 or a combination of both PD 184352 and SB203580 indicating that the initial induction of these miRNAs in response to LPS was independent of MAPK signaling (Fig. 6A). Unexpectedly SB203580 did result in a small increase in pri-miR-155, and to a lesser extent, pri-miR-455 levels. Similar trends were found after 24 h of LPS stimulation (data not shown) or following 4 h of stimulation with heat killed *C. albicans*, although the increases in pri-miR-155 and pri-miR-455 levels caused by SB203580 treatment were more pronounced following *C. albicans* treatment compared to LPS (Fig. 6B). A second difference between LPS and *C. albicans* was that pri-miR-146a induction was reduced by a combination of PD184352 and SB203580 following heat killed *C. albicans* but not LPS stimulation. LPS induced pri-miR-146b expression was however slightly reduced by SB203580 and greatly reduced by PD184352 (Fig. 6A). pri-miR-125a induction by LPS was also slightly decreased by pretreatment with either SB203580 or PD184352, but was greatly reduced by a combination of both PD184352 and SB203580. A similar trend on pri-miR-125a transcription was obtained following heat killed *C. albicans* stimulation (Fig. 6B), although the degree of inhibition by either PD184352 or SB203580 was greater following heat killed *C. albicans* than LPS stimulation.

### NFκB is required for LPS and *Candida albicans* induced miRNA induction

**Figure 7:**
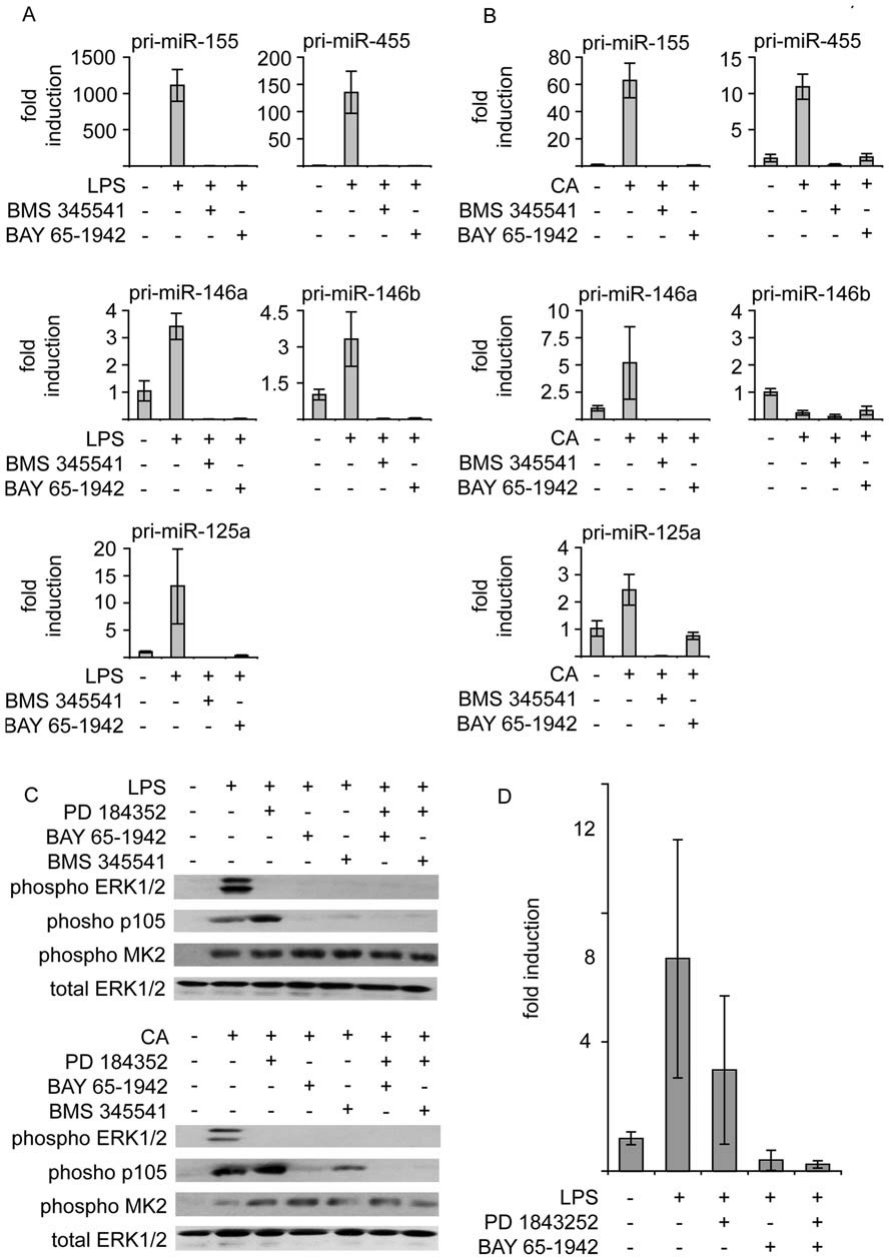
Effect of IKKβ inhibitors on the transcription of miR-155, miR-455, miR-146 and miR-125a. (A) BMDMs were treated for 1 h where indicated with 15 µM BMS 345541 or 10 µM BAY 65-1942. Cells were then stimulated with 100 ng/ml LPS for 1 h and total RNA isolated. The levels of pri-miR-155, pri-miR-455, pri-miR-146a, pri-miR-146b and pri-miR-125a were determined by Q-PCR. 18S was used as a loading control. Error bars represent the standard deviation of independent stimulations cultures from 4 mice. (B) As (A) except that cells were stimulated with 10^6^ cells/ml heat killed *C. albicans* for 4 h. (C) Where indicated BMDMs were pretreated with 2 µM PD 184352, 10 µM BAY 65-1942 or 15 µM BMS 345541 as indicated. Cells were stimulated with 100 ng/ml LPS or 10^6^ cells/ml heat killed *C. albicans* for 30 min and the levels of phospho ERK1/2, total ERK1/2, phospho MK2 and phospho p105 were determined by immunoblotting. (D) As (C) except that total RNA was isolated and the levels of pri-miR-125a determined by Q-PCR. Error bars represent the standard deviation of independent stimulations cultures from 4 mice.

Both LPS and heat killed *C. albicans* activate the NFκB pathway in macrophages. NFκB has been implicated in the induction of the transcripts encoding miR-155 and miR-146a [6], [19]. To determine the importance of NFκB in the induction of the selected primary miRNAs, BMDMs were pretreated with the IKKβ inhibitors BMS345541 or BAY65-1942. Both the LPS and heat killed *C. albicans* stimulated transcription of pri-miR-155, pri-miR-455, pri-miR-146a and pri-miR-125a as well as the LPS induced transcription of pri-miR-146b were reduced by pretreatment with the IKKβ inhibitors, suggesting that IKKβ plays a critical role in the induction of these miRNAs (Fig. 7A and B). Consistent with pri-miR-125 and pri-miR-455 being co-transcribed with Ncrna00085 and Col27a1 respectively, the induction of both these mRNAs was also blocked by IKKβ inhibitors (data not shown). For pri-miR-155, pri-miR-455 and pri-miR-146a this would suggest that NFκB plays a direct role in the transcription of these miRNAs. However, for pri-miR-125a and pri-miR-146b the situation is less clear, as IKKβ is involved in the activation of ERK1/2 in response to LPS, and induction of these miRNAs is reduced by inhibitors of the ERK1/2 pathway (Fig. 6). In response to TLRs, the ERK1/2 – MKK1/2 cascade is activated by the upstream kinase Tpl2. Tpl2 is normally held in an inactive complex with p105, a member of the NFκB family. Activation of Tpl2 requires the phosphorylation of p105 by IKKβ, which allows dissociation of the complex [20], [21]. Thus IKK inhibitors can block LPS or heat killed *C. albicans* stimulated ERK1/2 activation in macrophages (Fig. 7C). Neither inhibitor affected the activity of p38, as judged by phosphorylation of the p38 substrate MK2. As PD184352 reduced, but did not block, the induction of pri-miR-125a we were able to test the ability of IKKβ inhibitors to inhibit pri-miR-125a transcription when ERK1/2 activation was blocked by PD184352. This demonstrated that any residual pri-miR-125a induction in the presence of PD184352 was still blocked by the IKK inhibitors, indicating that IKKβ plays a role in miR-125a transcription independent of its ability to regulate ERK1/2 activation (Fig. 7D).

### IL-10 modulates miRNA production in macrophages

**Figure 8:**
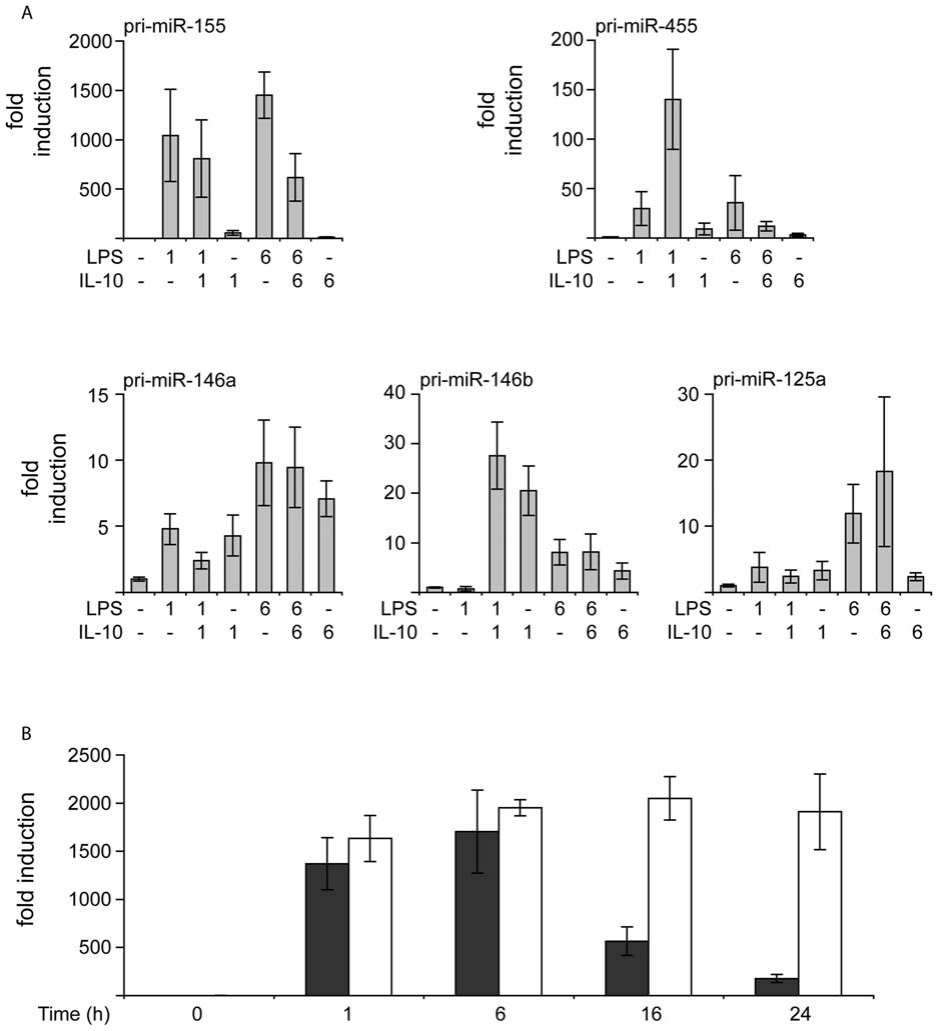
Regulation of miRNA by IL-10. A) BMDMs were stimulated with either 100 ng/ml IL-10, 100 ng/ml LPS or a combination of both IL-10 and LPS for 1 or 6 h. Total RNA was isolated, and the levels of pri-miR-155, pri-miR-455, pri-miR-146a, pri-miR-146b, pri-miR-125a were determined by Q-PCR. Error bars represent the standard deviation of independent cultures from 4 mice. B) BMDMs were cultured from either wild type (black bars) or IL-10 knockout (white bars) mice and stimulated with 100 ng/ml LPS for the indicated times. RNA was isolated and pri-miR-155 levels determined by Q-PCR. Error bars represent the standard deviation of independent cultures from 4 mice per genotype.

For both miR-155 and miR-146, their most likely role in macrophages is to act as a negative feedback mechanism to limit inflammatory signaling. IL-10 is an anti-inflammatory cytokine, which can limit inflammatory signaling in macrophages by inducing the STAT3 dependent transcription of anti-inflammatory genes [22]. Previous studies have shown that for several protein encoding genes, IL-10 can synergistically induce their transcription in combination with LPS [23]. We therefore tested the effect of IL-10, either alone or with LPS, on pri-miRNA transcription in BMDMs. Both pri-miR-146a and pri-miR-146b could be induced by IL-10 alone, however there was no major synergistic effect with LPS (Fig. 8A). IL-10 was a weak activator of pri-miR-125a transcription and there was no synergistic effect with LPS (Fig. 8A). In contrast, while IL-10 alone was a relatively weak stimulus for pri-miR-455 transcription, a combination of both IL-10 and LPS resulted in a much greater stimulation of pri-miR-455 than either LPS or IL-10 alone at 1 h, although this synergistic effect of LPS and IL-10 was not seen at 6 h (Fig. 8A). IL-10 alone had little effect of pri-miR-155 transcription, and did not affect LPS induced pri-miR-155 levels at 1 h, although at 6 h exogenous IL-10 was able to repress LPS induced pri-miR-155 levels (Fig. 8A). LPS stimulates IL-10 production from macrophages, and the released IL-10 can act on the macrophages in an autocrine manner. The LPS induced transcription of the pri-miR-155 was therefore measured in BMDMs from IL-10 knockout mice. At early time points following LPS stimulation, miR-155 levels were similar in wild type and IL-10 knockout cells, however at 16 and 24 h of LPS stimulation, IL-10 knockout cells expressed higher levels of pri-miR-155 (Fig. 8B).

## Discussion

We show here that miR-155, miR-146a, miR-146b, miR-125a and miR-455 can be up-regulated by the TLR4 agonist LPS as well as by heat killed *C. albicans* which most likely signals via a combination of TLRs and the C-type lectin dectin-1 [24], [25]. The heat killed yeast from of *C. albicans* was used in this study. *In vivo* it is normally the hyphal form of the fungus which is invasive. This coupled with the fact that the heat treatment may modify the cell wall structure, means that the ligands exposed on the surface of the heat killed *C. albicans* will not be completely identical to those seen in an *in vivo* infection. The induction of miR-155, miR-146a and miR-146b has been shown previously in response to viral and bacterial mimics that stimulate via a variety of TLRs [6], [7], [8], [10], [11], however this is the first study to look at their induction by fungal ligands. The regulation of miR-125a and miR-455 in response to either of these stimuli has not previously been examined.

Of the tested miRNAs, only miR-146a was located in an intragenic region. It has been shown that some intronic miRNAs are co-transcribed with the gene that contains them. miR-455 is encoded within an intron of the Col27a1 gene, which encodes type XXVII collagen. Similar to pri-miR-455, Col27a1 transcription was induced by LPS or heat killed *C. albicans*. This would be consistent with the processing of miR-455 from the intron of the Col27a1 transcript. miR-125a is located adjacent to Ncrna00085, and the regulation of pri-miR-125a and Ncrna00085 by LPS or heat killed *C. albicans* was similar suggesting they are co-transcribed. The function of Ncrna00085 is unknown; while it contains a potential open reading frame it is not clear if it is translated *in vivo*. An unusual feature of this locus is that pri-miR-125 actually overlaps an intron exon boundary, suggesting that processing of the miRNA would occur in competition with splicing of the mRNA. miR-125a is located in the genome within 1 kb of miR-99b and let-7e. In BMDMs no significant up-regulation of either miR-99b or let-7e in response to LPS or heat killed *C. albicans* was observed in this study. In contrast, a recent report showed that LPS could up-regulate let-7e in peritoneal macrophages and that this was dependent on Akt1 activity [26]. The reason for that difference is not clear, but could relate to the source of macrophages. In this respect it should be noted that LPS is only a weak activator of the Akt pathway in BMDMs (data not shown), and that cell type specific differences in response to LPS have been described for another miRNA, miR-132 [27].

All 5 of the tested pri-miRNAs were found to require NFκB for their induction in response to LPS or heat killed *C. albicans*. The transcription of miR-455 and miR-125a has not previously been studied in immune cells, while miR-146 has been reported to be an NFκB target gene. In humans, pri-miR-155 is encoded in the non-coding BIC gene [11], and potential AP-1 and NFκB elements have been identified in the promoter of this gene which are conserved in mice [11], [19], [28]. In B cells, transcription of BIC following stimulation of the B cell receptor has been shown to require the AP-1 elements in it's promoter [28]. AP-1 is a dimeric complex that can be formed from Jun or fos family members (reviewed in [29], [30]). AP-1 activity can be regulated by MAPK signaling – either via the transcriptional induction of immediate early genes such as c-fos and junB or via the direct phosphorylation of the AP-1 complex downstream of MAPK signaling. We find that in response to LPS, MAPK signaling does not directly regulate pri-miR-155 transcription, suggesting that regulation of AP-1 activity by LPS is not the major mechanism for inducing miR-155 production in macrophages. Our data does not rule out a requirement for AP-1 for basal miR-155 transcription in macrophages. We found however that inhibitors of IKKβ, which block NFκB activation, abolished pri-miR-155 induction by LPS or heat killed *C. albicans* suggesting that NKκB may be the main signaling input downstream of TLRs and dectin-1 that regulate pri-miR-155 transcription in macrophages.

IL-10 is known to modulate the transcription of many genes that are induced in response to the activation of PRRs, and to repress the inflammatory action of macrophages. Of the miRNAs tested, IL-10 alone was able to induce pri-miR-125a, pri-miR-146a and pri-miR-146b, however there was no synergy with LPS. IL-10 did however have a synergistic effect with LPS on pri-miR-455 induction, however this appeared to be restricted to early time points. In contrast, LPS induced pri-miR-155 transcription at later time points was inhibited by IL-10. In line with this, pri-miR-155 levels were higher at later time points after LPS stimulation in IL-10 knockouts relative to wild type cells. A similar finding has been published recently [31]. IL-10 represses pro-inflammatory cytokine production by macrophages. While it is possible that IL-10 may have a direct repressive effect on pri-miR-155 transcription, a 2^nd^ explanation could be that prolonged pri-miR-155 expression may require re-stimulation of the macrophages by pro-inflammatory cytokines in an autocrine manner. This autocrine feedback would be reduced by IL-10. miR-155 induction by TLR4 has recently been shown to be required in Akt1 knockouts [26]. Akt inhibition reduces IL-10 [32] and so a reduction in the IL-10 autocrine repression of miR-155 may explain the increased miR-155 levels in the Akt1 knockout.

miR-146 and miR-155 are reported to target proteins that are involved in inflammatory signaling, including IRAK1, Traf6 and Myd88, which would suggest that these miRNAs serve to limit the inflammatory capacity of the macrophages. The effects of these miRNAs on the activity of macrophages following fungal or bacterial stimuli would be expected to be similar, and thus the induction of specific miRNAs may provide a negative feedback loop to block excessive inflammation following fungal infection. Interestingly miR-155 has been shown to down-regulate DC-SIGN, a C-type lectin that recognizes mannose containing glycoproteins expressed by variety of pathogens including fungi [33]. It could be speculated that the significantly lower expression of miR-155 following heat killed *C. albicans* stimulation rather than LPS could help play a role in tailoring the macrophage response to the appropriate pathogen. The targets for miR-125a and miR-455 are not well established. *In silico* predictions by Target scan (http://www.targetscan.org) for miR-125a and miR-455 however suggest they may target some proteins involved in inflammatory signaling, including IRF4, MLK2, IL-1 receptor protein 1, OTBU2 and c-maf, suggesting that these miRNAs may also play a role in limiting inflammation. Further studies will however be required to delineate the role of these miRNA is the innate immune system and their importance in the response to fungal infection.

## Methods

### Cell culture

Bone marrow derived macrophages (BMDMs) were isolated from C57Bl/6 mice as described [34]. IL-10 knockout mice have been described previously [35]. Mice were maintained in accordance with UK and EU regulations, and work was covered by an appropriate home office license (60/3923) which was subject to review by the University of Dundee Ethical Review Committee. Cells were maintained on bacterial grade plates for 1 week in DMEM supplemented with 10 % heat inactivated FBS (Sigma), 2 mM L-glutamine, 100 units/ml penicillin G, 100 µg/ml streptomycin 0.25 µg/ml amphotericin (Invitrogen) and 5 ng/ml rCSF. Adherent cells were then replated on tissue culture plastic in fresh media and used 24 hours after replating.

Where indicated, cells were incubated in 5 µM SB 203580, 2 µM PD 184352, 25 µM SP 600125, 15 µM BMS 345541 or 10 µM BAY 65-1942 for 1 hour before use. Cells were stimulated with 100 ng/ml LPS (Sigma, L6529), 10^6^ cells/ml heat killed *C. albicans* (Invivogen) or 100 ng/ml IL-10 (R&D Systems) for the indicated times. Specificity profiles for these inhibitors have been published, and the compounds were used at minimum concentration that completely blocked activity of the target kinase in cells [18].

### Microarray

For microarray experiments RNA from BMDMs was isolated using the microRNeasy mini kits (Qiagen) in line with the manufacturer's protocol. Array profiling was carried out by LC Biosciences. Briefly, control and stimulated samples were labeled with either Cy3 or Cy5 and hybridised to arrays designed against murine miRNA in miRBase release 12. Data was analysed using ArrayPro software. Array data is deposited in GEO under the accession number GSE21970.

### Quantitative RT PCR

RNA was isolated using the microRNeasy mini kits (Qiagen) in line with the manufacturer's protocol. For analysis of mRNA or primary miRNA transcripts, total RNA was reverse transcribed using iScript (Biorad), and real time PCR carried out using Sybrgreen based detection methods. Primers for these PCRs are shown in table 3. For expression analysis 18S was used as a loading control and fold induction was determined from Ct values using the equation:



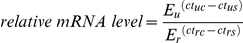



**Table 3:** Primer sequences used for Q-PCR.

Primer description	Sequence
pri-mmu--mir-146a sense	CACGGACCTGAAGAACACTGG
pri-mmu-mir-146a anti-sense	AGAAATGAAATTAGAACACACATCAATCC
pri-mmu-mir-146b sense	GGCAGCATCCAGACTGAGAG
pri-mmu-mir-146b anti-sense	GCCTTGGTGTTGATGGTATAGC
pri-mmu-mir-155 sense	ACTAGCACTCACATGGAACAAATGG
pri-mmu-mir-155 anti-sense	CCAGGTTATGACTAGCACATTAAATGATAG
Pri-mmu-miR455 sense	CCTGGGTACGAGCTTCCTTCC
Pri-mmu-miR455 anti-sense	GCTGCGTTCACGATGTAGTCC
Pri-mmu-miR-125a sense	TCCCTGAGACCCTTTAACC
Pri-mmu-miR-125a anti-sense	TCACCTGAAATCCCTAAATTTG
Ncrna00085 exon 9 sense	CTGACGCTTGGCAACCTATTCC
Ncrna00085 exon 9 anti-sense	GCTGTTGAGGTGGTGACAAAGG
Ncrna00085 intron 1 sense	CTCTAGCTGGAGCCTGATGC
Ncrna00085 intron 1 anti sense	CGGGTCTGAGGAGAAGATAGTG
Ncrna00085 exon1 1-2 spanning sense	ATCTGTTTCTGTCTCGCTTCCC
Ncrna00085 exon 1-2 spanning anti-sense	GACAATGCTCCCTACTGTGACC
Tmem 180 exon 4 sense	CTCCTCCTTCCGTGCCTTCTG
Tmem 180 exon 4 anti-sense	CCTTCTGGTCGCTTCAATCTGC
Col27a sense	AGACCTGACAACCAAGCCTAGC
Col27a anti-sense	TCAGCATCGGGAAAGGTGTAGG
18S sense	GTAACCCGTTGAACCCCATT
18S anti-sense	CCATCCAATCGGTAGTAGCG

where E is the efficiency of the PCR, ct is the threshold cycle, u is the mRNA of interest, r is the reference gene (18S RNA), s is the sample and c is the unstimulated control sample.

Q-PCR for mature miRNA was carried out using TaqMan MiRNA assays from Applied Biosystems according to the manufacturer's protocols. 18S levels were used to correct for total RNA levels.

### Immunoblotting

Cells were lysed in 1% (w/v) SDS, 10% (v/v) glycerol, 50 mM Tris–HCl pH 7.5, 1 mM EGTA, 1 mM EDTA, 1 mM sodium orthovanadate, 50 mM sodium fluoride, 1 mM sodium pyrophosphate, 0.27 M sucrose, 1% (v/v) Triton X-100, 0.1% (v/v) 2-mercaptoethanol. Samples were run on 10 % polyacrylamide gels, and immunoblotted using standard techniques. Antibodies against phospho ERK1/2, total ERK1/2, phospho p38, total p38, phospho JNK, phospho p105, phoshpho MK2 and total IκB were from Cell Signalling Technology. HP-conjugated secondary antibodies were from Pierce, and detection was performed using the enhanced chemiluminescence reagent from Amersham.
